# Aggressive behavior and prognosis in patients with mild cognitive impairment

**DOI:** 10.1590/1980-5764-DN-2020-0096

**Published:** 2023-04-14

**Authors:** Leszek Bidzan, Jakub Grabowski, Mateusz Przybylak, Shan Ali

**Affiliations:** 1 Medical University of Gdańsk, Faculty of Medicine, Department of Developmental, Psychotic and Geriatric Psychiatry, Gdańsk, Poland.; 2 Medical University of Gdańsk, Faculty of Medicine, Department of Developmental, Psychotic and Geriatric Psychiatry, Adult Psychiatry Student’s Scientific Circle, Gdańsk, Poland.

**Keywords:** Dementia, Neurocognitive Disorders, Impulsive Behavior, Aggression, Behavioral Symptoms, Cognitive Dysfunction, Demência, Transtornos Neurocognitivos, Comportamento Impulsivo, Agressão, Sintomas Comportamentais, Disfunção Cognitiva

## Abstract

The diagnosis of mild cognitive impairment (MCI) is associated with an increased risk of developing dementia. When evaluating the further prognosis of MCI, the occurrence of neuropsychiatric symptoms, particularly aggressive and impulsive behavior, may play an important role.

**Objective:**

The aim of this study was to evaluate the relationship between aggressive behavior and cognitive dysfunction in patients diagnosed with MCI.

**Methods:**

The results are based on a 7-year prospective study. At the time of inclusion in the study, participants, recruited from an outpatient clinic, were assessed with Mini-Mental State Examination (MMSE) and the Cohen-Mansfield Agitation Inventory (CMAI). A reassessment was performed after 1 year using the MMSE scale in all patients. The time of next MMSE administration was depended on the clinical condition of patients took place at the end of follow-up, that is, at the time of diagnosis of the dementia or after 7 years from inclusion when the criteria for dementia were not met.

**Results:**

Of the 193 patients enrolled in the study, 75 were included in the final analysis. Patients who converted to dementia during the observation period exhibited a greater severity of symptoms in each of the assessed CMAI categories. Moreover, there was a significant correlation between the global result of CMAI and the results of the physical nonaggressive and verbal aggressive subscales with cognitive decline during the first year of observation.

**Conclusions:**

Despite several limitations to the study, aggressive and impulsive behaviors seem to be an unfavorable prognostic factor in the course of MCI.

## RESUMO

O diagnóstico de comprometimento cognitivo leve (CCL) está associado a um risco aumentado de desenvolver demência. Ao avaliar o prognóstico adicional do CCL, a ocorrência de sintomas neuropsiquiátricos, particularmente o comportamento agressivo e impulsivo, pode desempenhar um papel importante.

**Objetivo:**

Avaliar a relação entre comportamento agressivo e disfunção cognitiva em indivíduos com diagnóstico de CCL.

**Métodos:**

Nossos resultados são baseados em um estudo prospectivo de sete anos. No momento da inclusão no estudo, os participantes, recrutados em um ambulatório, foram avaliados com o Mini-Exame do Estado Mental (MEEM) e o Inventário de Agitação de Cohen-Mansfield (CMAI). A reavaliação foi realizada após um ano com a escala MEEM em todos os pacientes. O momento da próxima administração do MEEM dependeu da condição clínica dos indivíduos e ocorreu no final do acompanhamento, ou seja, no momento do diagnóstico da demência ou após sete anos da inclusão, quando os critérios para demência não foram atendidos.

**Resultados:**

Dos 193 pacientes incluídos no estudo, 75 foram incluídos na análise final. Os indivíduos que converteram para demência durante o período de observação exibiram uma maior gravidade dos sintomas em cada uma das categorias avaliadas pelo CMAI. Além disso, houve uma correlação significativa entre o resultado global do CMAI e os resultados das subescalas de agressão física e verbal com declínio cognitivo durante o primeiro ano de observação.

**Conclusões:**

Apesar das várias limitações do estudo, os comportamentos agressivos e impulsivos parecem ser um fator prognóstico desfavorável no curso do CCL.

## INTRODUCTION

Mild cognitive impairment (MCI) is considered an intermediate condition between physiological aging and dementia. In MCI, neuropathological processes are already occurring and disturbances in cognitive abilities may be observed^1,2,3^. The prevalence of MCI in the elderly population is about 15–20%^4^, and MCI is classified as a definite risk factor for the development of Alzheimer’s dementia (AD)^5,6,7,8^. A study shows that, over 5 years, more than 50% of people diagnosed with MCI will fully develop symptomatic dementia^9^. Yet, many patients diagnosed with MCI do not experience further impairment of cognitive functions and the diagnosis of MCI does not necessarily determine the prognosis of a patient^10,11^.

The neuropsychiatric symptoms of MCI may manifest as anxiety and restlessness, depressed mood, sleep disorders, psychotic symptoms, aggressive or impulsive behaviors, apathy, agitation, disinhibition, psychosis, irritability, euphoria, or aberrant motor behaviors^12–14^. Out of the many symptoms, aggressive behavior is associated with a faster progression of cognitive disorders^15–17^. Therefore, physicians often pay particular attention to the prognostic significance of aggression and the advancement of dementia^18,19,20^.

To the best of our knowledge, no study has yet evaluated the relationship between aggressive behavior and cognitive dysfunction in patients diagnosed with MCI. The definition of aggression itself is ambiguous as it describes diverse forms of behavior. Not all types of aggressive and impulsive behaviors are likely to show a relationship with cognitive impairment. Our aim was to assess the relationship between aggression and impulsive behavior with cognitive dysfunctions. To perform this, we conducted a 7-year prospective study on people diagnosed with MCI.

## METHODS

This study is a continuation of research examining the prognostic factors in MCI and contains similar material and methods^21,22^. Parts of the tables describing population characteristics and the Mini-Mental State Examination (MMSE) scores used in this study are already published under the terms of the Creative Commons Attribution-NonCommercial-Share Alike 4.0 International with allowance for copy and redistribution^21^. This study received approval by the Independent Bioethics Commission for Research at the Medical University of Gdansk, in Gdansk, Poland (NKEBN/377/2009).

### Inclusion and exclusion criteria

All patients and their caregivers signed the informed consent forms. A full psychiatric examination was carried out on all the qualified patients and included the MMSE and the CMAI. We included 193 patients in the study. They were recruited at the Mental Health Clinic (an outpatient clinic) in northern Poland from 2005 to 2007. These patients underwent a systematic psychiatric observation during routine ambulatory visits, every two to three visits on average, to the point of dementia diagnosis over the 7-year observation period. In the course of the visits, all patients were applied to clinical expert evaluation. Patients who were qualified had MCI diagnosed based on the three-part Working Group on MCI criteria that consist of three basic parts:

- The exclusion of dementia,
- Evidence of cognitive decline based on the patient’s self-assessment and/or caregiver’s information with confirmation of cognitive impairment on objective cognitive tasks and/or evidence of objective cognitive decline over time, and
- Basic activities of daily living were preserved with a possible minimal impairment in complex instrumental functions.

The examination was necessary for the caregiver who lived with the patient or visited him several times a week and was willing to participate in Cohen-Mansfield Agitation Inventory (CMAI).

### Assessment of aggressive behavior

Assessment of agitation and aggressive behavior was based on the CMAI that was designed for people with cognitive impairment^23^. In this study, a 29-element tool was used. Individual behavior types included in the inventory are attributed to four dimensions: verbal nonaggressive, physical nonaggressive, verbal aggressive, and physical aggressive. The validated Polish-language version of CMAI was used to increase the credibility of the results, since the study was conducted on Polish-native patients^24^. Although the original version of CMAI was prepared for the assessment of people living in nursing homes, the attached scale instructions in application was also verified positive in populations living outside stationary care units^25^.

### Assessment of cognitive function

Assessment of cognitive functions was based on the MMSE scale and was performed again in the study group 1 year (within 9–13 months) after baseline. The next MMSE took place at the end of observational period (i.e., either at the diagnosis of dementia or 7 years after enrollment).

The patients included in the study were under the direct supervision of psychiatrists (the authors of this study) and their mental state was systematically monitored several times a year. The diagnosis of psychiatric disorders was based on the *Diagnostic and Statistical Manual of Mental Disorders IV Text Revision* criteria^26^. On each psychiatric consultation, the clinical diagnosis was verified, especially when confirming or excluding dementia. When the diagnosis of a dementia syndrome was being confirmed, laboratory tests and other examinations to establish the etiology of the process were carried out. Therefore, all patients who met criteria of dementia underwent routine protocol of additional examinations consisting of neuroimaging test (computed tomography) and basic laboratory tests as complete blood count, glucose, triglycerides, cholesterol, and alanine and aspartate aminotransferases.

### Statistical methods

Statistical methods were based on parametric tests (Student’s t test for two independent means). A p-value ≤ 0.05 was considered significant. A two-sided confidence interval (CI) was assumed. The assessment of cognitive functions was based on the MMSE had its obtained score (raw results) were recalculated based on the formula proposed by Mungas et al.^27^, which was previously verified in Polish patients by Józwiak et al.^28^. Corrected results were used for statistical analysis.

## RESULTS

Of the 193 patients enrolled in the study, 75 were included in the final analysis, since they completed the 7-year observation period or were diagnosed with dementia during that time. Dementia developed in 34 patients, in particular, 16 AD, 4 vascular dementia, 2 Lewy bodies dementia, 3 frontotemporal dementia, and 9 mixed or unknown etiology dementia. The types of dementia were not analyzed (due to the insufficient size of the study group and the relatively large group of people who did not have a definitive etiological diagnosis).

Based on the final diagnosis, patients were grouped based on the presence of stable MCI (MCI-S) or converting MCI (MCI-C). Thirty-four patients had MCI-C, since they had conversion to dementia during the 7-year follow-up. Forty-one patients had MCI-S, since they had no basis to be diagnosed with dementia during the 7-year follow-up.

Patients with MCI-C during the follow-up period showed a greater severity of aggressive and impulsive behavior at baseline when considering the overall result of the CMAI scale and its subcategories. Both MCI-S and MCI-C groups presented only a slight intensity of aggressive behaviors; the global CMAI scale for each group was 62.79 and 43.78, respectively, as presented in Table 1. The result is low when considering the scoring range (29–203).

**Table 1.** Comparison of age, mean Mini-Mental Scale Examination results, and Cohen-Mansfield Agitation Inventory results at baseline in group of patients with mild cognitive impairment which either converted to dementia (n=34) or had a stable course (n=41).

Value	MCI-C Mean	MCI-S Mean	t	df	p	MCI-C SD	MCI-S SD	F	p-var.
Age (years)	78.79	75.76	1.44	73	0.16	8.40	9.67	1.33	0.41
MMSE Ic	27.20	27.45	-0.36	73	0.72	3.08	2.89	1.13	0.70
MMSE IIc*	24.70	26.33	-2.28	73	0.03	3.37	2.83	1.42	0.29
MMSE I–IIc*	2.50	1.12	2.82	73	0.01	2.03	2.17	1.14	0.71
MMSE VIIc*	19.14	26.06	-7.05	73	<0.01	5.40	2.93	3.39	0.00
CMAI*	62.79	43.78	5.62	73	<0.01	13.75	15.23	1.23	0.55
VNA*	13.21	9.51	3.16	73	<0.01	4.23	5.61	1.76	0.10
PNA*	26.18	15.24	6.79	73	<0.01	6.85	7.02	1.05	0.89
VA*	8.94	6.17	3.11	73	<0.01	4.57	3.10	2.18	0.02
PA*	14.47	12.85	4.17	73	<0.01	1.48	1.81	1.49	0.24

*Statistical significance (p<0.05). MMSE: Mean Mini-Mental Scale Examination Low; MCI-C: patients with primary mild cognitive impairment that converted to dementia; MCI-S: patients with stable course of mild cognitive impairment; t: Student’s t test distribution; df: degrees of freedom; SD: standard deviation; F: analysis of variance by Fisher-Snedecor distribution; p-var.: variances of statistical significance; CMAI: Cohen-Mansfield Agitation Inventory results; VNA: CMAI verbal nonaggressive subscale results; PNA: CMAI physical nonaggressive subscale results; VA: CMAI verbal aggressive subscale results; PA: CMAI physical aggressive subscale results.

Tables 1–4. present, respectively, the mean values of age and scale scores obtained for the studied patient population included in the final analysis (Table 2), a comparison of the results obtained at baseline in groups of people with converting and stable MCI (Table 1), a comparison of results obtained at baseline in groups with MCI with higher or lower MMSE score at inclusion (mean MMSE score = 27.34 taken as a border value) (Table 3), and a comparison of results with respect to difference in scoring between the first and second MMSE measurements in patients with MCI (mean difference between scores MMSE I–IIc=1.75 taken as a border value) (Table 4).

**Table 2.** The Mean Mini-Mental Scale Examination and Cohen-Mansfield Agitation Inventory values of patients included in the final analysis.

Value	Mean	Minimum	Maximum	Standard deviation
Age (years)	77.13	57.00	96.00	9.18
MMSE Ic	27.34	21.55	33.50	2.96
MMSE IIc	25.59	18.51	31.80	3.17
MMSE I–IIc	1.75	−4.00	6.00	2.21
MMSE VIIc	22.92	5.00	31.19	5.45
CMAI	52.40	30.00	78.00	17.34
VNA	11.19	4.00	21.00	5.33
PNA	20.20	11.00	33.00	8.81
VA	7.43	4.00	17.00	4.05
PA	13.59	11.00	16.00	1.85

MMSE: Mean Mini-Mental Scale Examination; MMSE Ic: MMSE score at baseline; MMSE IIc: MMSE score after 1 year (on the second examination); MMSE I–IIc, difference between MMSE score on the first and second examination; MMSE VIIc: MMSE score at the end of study (in the seventh year of observation or at the moment of dementia diagnosis); CMAI: Cohen-Mansfield Agitation Inventory results; VNA: CMAI verbal nonaggressive subscale results; PNA: CMAI physical nonaggressive subscale results; VA: CMAI verbal aggressive subscale results; PA: CMAI physical aggressive subscale results.

**Table 3.** Comparison of age, Mean Mini-Mental Scale Examination results, and Cohen-Mansfield Agitation Inventory results at baseline in group of patients with mild cognitive impairment divided in two groups — those with a higher Mean Mini-Mental Scale Examination score (n=41) and a lower Mean Mini-Mental Scale Examination score (n=34); the mean Mean Mini-Mental Scale Examination score=27.34 was established as the threshold.

Value	Low MMSE Ic Mean	High MMSE Ic Mean	t	df	p	Low MMSE Ic SD	High MMSE Ic SD	F	p-var.
Age (years)*	75.17	79.50	-2.08	73.00	0.04	9.89	7.74	1.63	0.15
MMSE Ic*	25.09	30.05	-13.19	73.00	<0.01	1.52	1.74	1.30	0.42
CMAI	54.80	49.50	1.33	73.00	0.19	17.15	17.37	1.03	0.93
VNA	11.68	10.59	0.88	73.00	0.38	5.10	5.63	1.22	0.55
PNA	21.39	18.76	1.29	73.00	0.20	8.69	8.86	1.04	0.90
VA	7.88	6.88	1.06	73.00	0.29	4.27	3.76	1.29	0.46
PA	13.85	13.26	1.38	73.00	0.17	1.77	1.91	1.17	0.63

*Statistical significance (p<0.05). MMSE: Mean Mini-Mental Scale Examination Low; MMSE Ic: MMSE score lower than the threshold (MMSE=27.34) at baseline; High MMSE Ic: MMSE score higher than the threshold (MMSE=27.34) at baseline; t: Student’s t test distribution; df: degrees of freedom; SD: standard deviation; F: analysis of variance by Fisher-Snedecor distribution; p-var.: variances of statistical significance; CMAI: Cohen-Mansfield Agitation Inventory results; VNA: CMAI verbal nonaggressive subscale results; PNA: CMAI physical nonaggressive subscale results; VA: CMAI verbal aggressive subscale results; PA: CMAI physical aggressive subscale results.

**Table 4.** Comparison of age, difference in Mean Mini-Mental Scale Examination scores over time and Cohen-Mansfield Agitation Inventory scores at baseline in group of patients with mild cognitive impairment in two groups — those with a higher (n=42) and a lower (n=33) difference in the Mean Mini-Mental Scale Examination score.

Value	High MMSE I–IIc Mean	Low MMSE I–IIc Mean	t	df	p	Low MMSE I–IIC SD	High MMSE I–IIc SD	F	p-var.
Age (years)	76.86	77.48	−0.29	73.00	0.77	7.83	10.78	1.90	0.05
MMSE I–IIc*	3.29	−0.21	11.09	73.00	<0.01	1.29	1.43	1.22	0.54
CMAI*	56.71	46.91	2.52	73.00	0.01	17.80	15.28	1.36	0.38
VNA	11.90	10.27	1.32	73.00	0.19	5.25	5.38	1.05	0.87
PNA*	22.60	17.15	2.78	73.00	0.01	8.75	8.01	1.19	0.61
VA*	8.31	6.30	2.18	73.00	0.03	4.51	3.11	2.10	0.03
PA	13.90	13.18	1.71	73.00	0.09	1.87	1.76	1.13	0.72

*Statistical significance (p<0.05). MMSE: Mean Mini-Mental Scale Examination; High MMSE I–IIc: difference in MMSE score higher than the threshold (MMSE I–IIc=1.75); Low MMSE Ic: difference in MMSE score lower than the threshold (MMSE I–IIc=1.75); t: Student’s t test distribution; df: degrees of freedom; SD: standard deviation; F: analysis of variance by Fisher-Snedecor distribution; p-var.: variances of statistical significance; CMAI: Cohen-Mansfield Agitation Inventory results; VNA: CMAI verbal nonaggressive subscale results; PNA: CMAI physical nonaggressive subscale results; VA: CMAI verbal aggressive subscale results; PA: CMAI physical aggressive subscale results.

## DISCUSSION

### Key findings

We found that aggressive and impulsive behavior constitutes a less favorable prognosis in patients with MCI. This significant relationship may assist clinicians to help predict the course of cognitive disorders and facilitate more effective discovery, prevention, and treatment strategies. Our article is novel as it is one of the few to evaluate the relationship between aggressive behavior and the prognosis in patients with MCI.

### Context

Our finding concerning a worse prognosis in patients with MCI that display aggressive behavior is supported in part by previous scientific findings. Two studies made a similar observation in the study of people with clinical forms of dementia^29,30^. In previous studies on dementia disorders, prognostic significance varied depending on the form of aggression and impulsivity^31,32^.

The mean result of the global CMAI scale for each group was low. This is understandable, as people with MCI are more frequently affected by mood disorders symptoms, with lesser intensity of aggressive and impulsive behavior, which is more typical for people diagnosed with dementia^33^. However, despite the relatively small intensity of aggressive and impulsive behaviors in the group of people with MCI, the results may suggest a relationship between them and further progression of cognitive decline. The above result is consistent with previously obtained in studies on population of people with AD^15,16^.

Occurrence of neuropsychiatric symptoms, such as apathy, irritability, and attention deficit disorders, was more frequent in the preclinical period of dementia^34^. Similar observations were made in studies on people diagnosed with MCI^11,13^. It was noted that the presence of some of these symptoms, especially aggressive behaviors and psychotic disorders, may be associated with a greater progression of dementia^35^. Specifically, different forms of agitation have been indicated to precede a faster progression of cognitive disorders^16^. A similar relationship also appears in the course of MCI. Results presented in Table 4 point to a difference in terms of occurrence of aggressive and impulsive behaviors depending on cognitive function disorders progression degree determined by the MMSE score during the first year of observation. Obtained results correspond with other studies on patients diagnosed with MCI, where it was suggested that the process of conversion from MCI to dementia is accelerated through the presence of neuropsychiatric symptoms^10,29,30,36^. However, in the light of other research, a reverse relationship seems more likely. A more rapid neurodegenerative process is responsible for more frequent occurrence of some noncognitive symptoms^35^.

Moreover, while MCI is perceived, in a sense, as an intermediate step between physiological aging and dementia, discrete structural anomalies of the central nervous system may be expected^37,38^. In a series of studies, structural changes in people with MCI were revealed in magnetic resonance imaging^39,40^. Similarly to the case of histochemical elements, observed neuroimaging abnormalities in people with MCI are of intensity between those of patients without any lesions and those with dementia and are usually found in crucial areas for the assessment of early stages of AD (hippocampus and olfactory cortex)^41^. In addition, prospective studies indicate that the reduction of hippocampus is related to a higher risk of conversion to dementia^42,43^. Similarly, although to a lesser extent, abnormalities in a number of other brain regions may suggest a higher risk of progression^44,45,46^. The changes in the above areas are recognized as having a pathogenetic significance in the occurrence of neuropsychiatric symptoms in the course of dementia, especially of primary degenerative etiology, with AD in the lead^47,48^. In the course of our study, the neuroimaging tests were not provided at the baseline (point of inclusion), neither further routinely in case of the absence of dementia syndrome. Therefore, the results are based on clinical pictures and prospective observation of psychopathological features without concern of possible abnormalities in brain structures, what seems to be important to precise correlation between neuropsychiatric symptoms and neurodegenerative process.

### Limitations

The first limitation of the present study is an exclusion of majority of patients (n=118) from the baseline due to the used rigid criteria in the final analysis. Such course of an investigation could be considered selection bias.

Our study is based on the assessment of cognitive function disorders on the result of the MMSE. This scale lacks sensitivity and it also does not enable precise assessment of separate cognitive domains. However, the aim of the undertaken research was not the assessment of individual areas, but the overall evaluation of cognitive functioning. First and foremost, the aim was to translate the results of MCI research into a more practical area. Tools that could be used in everyday clinical work in a significant number of patients were used. While the MMSE is possible to be widely used in outpatient practice, other more complex, extensive, and thus time-consuming methods of studying cognitive functions may not be practical for mass adoption. It is worth noting that, despite the use of a “simple” MMSE, differences in progression of cognitive dysfunctions during the first year of observation effectively discriminated patients with MCI-C and MCI-S.

Moreover, the diagnosis of dementia in the study was based on the *Diagnostic and Statistical Manual of Mental Disorders IV Text Revision* criteria, according to which memory impairment criterion (A1) and the impairment of at least one another cognitive domain such as aphasia, apraxia, agnosia, or impairment of executive function (A2) are mandatory to be present^26^. Simultaneously, behavioral and personality changes are not considered as diagnostic criteria. In an investigation that evaluates behavioral symptoms and progression to dementia, using the criteria that require memory impairment is an unequivocal, diagnostic bias. There is a possibility that patients with dementia have not been included in the MCI-C group because of the absence of memory impairment. This subgroup would probably have performed better in the MMSE score, despite having dementia according to the current criteria.

Although at the time of inclusion in the study, the participants did not take psychotropic drugs, at least some of them (n=39) took such medications during the first year of observation, a period significant for the analysis of obtained results. Thus, concomitant psychiatric treatments were not completely taken into account. The reason for administration of such treatment was usually anxiety, depressed mood, sleep disorders, but also in some cases aggressive and impulsive behavior. In every particular case mentioned psychopathological symptoms did not meet diagnostic criteria of mental disorder, other than MCI (if they met, it would become reason of exclusion of further clinical observation). The medications used were valproic acid (9 patients), sertraline (7 patients), risperidone (5 patients), quetiapine (15 patients), and tianeptine (16 patients). Total number of medications usage is higher than the number of patients who undergone pharmacotherapy, because in some cases there were need to modify primarily supplied psychotropic drugs. Although it is not possible to rule out the influence of these drugs on the neurodegeneration mechanisms of brain, their potential impact on the results obtained in the assessment of cognitive functions (i.e., the MMSE) and aggressive or impulsive behaviors seems more significant. The study was of an observational nature, which made it impossible to discontinue treatment preceding the assessment of cognitive functions.

### Future directions

Aggression is an extremely complex phenomenon, conditioned by several factors. Other causes, which may affect aggressive and impulsive behavior, such as social factors, may be evaluated in future studies. Moreover, confirmatory studies need to be performed to confirm the conclusions of our study.

At present, the precise of MCI remains ambiguous; especially when concerning the differentiation of MCI *per se* from preclinical periods of dementia (and its various forms). Therefore, we encourage physician-scientists to create a narrower and more robust concept of MCI, since it is currently a broad term that covers various heterogeneous states.

Despite some limitations, we conclude that a higher incidence and severity of neuropsychiatric disorders, especially aggressive and impulsive behaviors, should be considered one of the unfavorable prognostic elements in the patients with MCI. The findings of this study may assist physicians to forecast the further course of observed cognitive disorders.
